# New Caledonian crows infer the weight of objects from observing their movements in a breeze

**DOI:** 10.1098/rspb.2018.2332

**Published:** 2019-01-09

**Authors:** Sarah A. Jelbert, Rachael Miller, Martina Schiestl, Markus Boeckle, Lucy G. Cheke, Russell D. Gray, Alex H. Taylor, Nicola S. Clayton

**Affiliations:** 1 Department of Psychology, University of Cambridge, Cambridge, UK; 2 School of Psychology, University of Auckland, Auckland, New Zealand; 3 Max Planck Institute for the Science of Human History, Max Planck Society, Jena, Germany; 4 Department of Psychotherapy, Bertha von Suttner University, St Pölten, Austria

**Keywords:** object properties, observational learning, causal reasoning, motion, corvid cognition

## Abstract

Humans use a variety of cues to infer an object's weight, including how easily objects can be moved. For example, if we observe an object being blown down the street by the wind, we can infer that it is light. Here, we tested whether New Caledonian crows make this type of inference. After training that only one type of object (either light or heavy) was rewarded when dropped into a food dispenser, birds observed pairs of novel objects (one light and one heavy) suspended from strings in front of an electric fan. The fan was either on—creating a breeze which buffeted the light, but not the heavy, object—or off, leaving both objects stationary. In subsequent test trials, birds could drop one, or both, of the novel objects into the food dispenser. Despite having no opportunity to handle these objects prior to testing, birds touched the correct object (light or heavy) first in 73% of experimental trials, and were at chance in control trials. Our results suggest that birds used pre-existing knowledge about the behaviour exhibited by differently weighted objects in the wind to infer their weight, using this information to guide their choices.

## 1. Introduction

Humans are able to use a variety of cues to infer the approximate weight of an object without direct handling. Using our prior experience, we can tell from glancing at the material that a piece of cardboard should be lighter than a plank of wood; that a ball of cotton wool should be lighter than a brick. We can also acquire information about the weight of unfamiliar objects through observing their physical interactions in the world. For example, if a human observes someone easily raising one box over their head, but then struggling to lift a second, the observer can immediately infer which box contains something heavy. By age five, children infer weight in this manner, judging an object's weight just by observing an actor lifting and transporting it [1], while adults are highly proficient at inferring an object's weight from observing another person's actions [2–4].

We can equally infer the weight of objects from observing their physical interactions with other inanimate objects, or with unobservable forces such as the wind. For example, if we see one cardboard box blowing down the street in a breeze, and one remaining stationary despite gale force winds, we can infer which box is heavy and which is light. This ability to draw inferences about an object's properties through observation, rather than only through direct handling, is likely to be useful in a wide range of contexts. It allows us to evaluate risks without encountering the potential danger directly [5], to anticipate the physical exertion required to lift an object [6,7], and to make complex judgements about concepts such as stability and support, even from infancy [8]. Given these wide-ranging uses, this ability may be shared with other species. Yet, at present, we know almost nothing about the extent to which non-human animals make inferences about properties of objects, such as weight, through observation alone.

A few studies have investigated the perception of weight by animals, focusing largely on chimpanzees. Povinelli argues, from several experiments conducted with chimpanzees, that only humans are capable of understanding directly unobservable causal features like weight [9]. They report that, while chimpanzees succeed at sorting objects on the basis of their weight, they fail to demonstrate the more sophisticated understanding of weight *as a causal force* in numerous contexts. This included experiments where the chimpanzees observed humans struggle to pull heavy boxes towards themselves, but failed to select a lighter box when given a choice, and experiments in which the chimpanzees heard the differing sounds of heavy and light objects being dropped behind a screen, but did not appear to expect the dropped object to have a particular weight. Other researchers argue that, like many cognitive abilities, any distinction between human and non-human capacities to understand weight is likely to be more nuanced [10,11], citing results that hint at a more comprehensive understanding of weight, found in other labs. For example, Hanus & Call demonstrated that chimps will search for a hidden banana in the lower of two cups on a balance beam [12], presumably by making the inference that the weight of the banana caused the balance beam to tilt. This experiment has been replicated in the aforementioned Povinelli study, which concluded that it is unfit to test for such understanding of weight without including a control condition involving a heavier weight, which the chimpanzees failed [9]. As yet, there is no clear consensus on the extent to which non-human primates perceive weight in a comparable manner to humans.

Studies with other species, including some corvids [13–16], indicate they will select nuts and seeds based on weight, probably as a proxy to distinguish between full and empty nuts before attempting to open them. However, to our knowledge, no experiments have investigated whether these species make inferences about objects' weights before directly handling them. This lack of research is striking given the wealth of investigations of core knowledge using observational paradigms with non-human animals. Core knowledge refers to the systems to represent objects, actions, number and space, possessed by humans, that emerge during typical development, and may be present from birth [17]. Among birds, research shows that precocial chicks enter the world with some core knowledge about objects, spatial relationships and number, as they will take into account spatial and numerical configurations when imprinting on artificial objects [18]. Research using expectation of violation paradigms has indicated that a variety of species (including corvids: rooks [19] and Eurasian jays [20]) possess an understanding of support relations—that objects will fall if they are not adequately supported by a solid surface—with rooks behaving similarly to 6.5-month-old humans.

In the current experiment, we tested whether one corvid species—New Caledonian crow (*Corvus moneduloides*)—makes judgements about weight through observation alone. We chose to focus on this species because they show sophisticated tool use and manufacture in the wild [21], and captive experiments demonstrate that these birds exhibit flexible manufacturing abilities [22,23] and understand some of the functional properties of their tools [23,24]. Like other corvids, they perform well on physical problem-solving tasks, such as the trap-tube [25,26] and Aesop's fable water displacement tasks [27–30]. New Caledonian crows may be a particularly promising test case because these birds have also demonstrated some capacity to make inferences in captivity. For example, they appear to be able to reason by exclusion [31], and about hidden causal agents [32]. Furthermore, when tested in object choice tasks, they readily discriminate between small objects of different weights (e.g. 1 versus 10 grams) [28,33,34], and recently, a study of the crows' exploration behaviour demonstrated that these birds learn about the object weight during their own spontaneous object exploration. The crows appeared to recall which objects they had experienced as light or heavy when later presented with an apparatus that could only be opened by dropping a heavy object inside [35]. However, crows failed to demonstrate an immediate understanding of the relevance of weight when dropping non-functional feathers [36], or other light objects [37], onto a collapsible platform.

Here, we designed a novel experiment to test whether wild-caught New Caledonian crows infer the weight of novel objects after observing their movements in a breeze, by assessing whether they use that information to guide their choices on an object-choice task. Specifically, crows were given the opportunity to observe novel objects being buffeted in a breeze created by an electric fan (light objects), or remaining stationary despite the breeze (heavy objects). To test this, crows were first trained that either heavy or light familiar objects were rewarded when dropped into a tube next to a food dispenser. Once they had acquired this rule, the birds were given the opportunity to observe novel objects suspended from strings in front of the fan, which was turned on and generating a breeze (experimental condition) or turned off (control condition). If birds had inferred the weight of the objects, when we later gave them the choice of the two novel objects, we expected them to be more accurate at selecting the correct object (light for six birds and heavy for six birds) when they had observed the objects moving in front of the fan when turned on, than in the control condition when the fan was off. If successful this would suggest the New Caledonian crows have a representation of weight as an unobservable causal mechanism—an ability that some researchers have claimed to be unique to humans [9].

## 2. Methods

### (a) Subjects

Subjects were 12 New Caledonian crows caught from the wild (at location 21.67° S, 165.68° E) on Grande Terre, New Caledonia. The birds were held in captivity in a large 10-compartment outdoor aviary, with approximately 7 × 4 × 4 m per compartment, on Grande Terre close, situated close to their location of capture, for the purposes of non-invasive behavioural research from April to August 2016 (six birds) and April to August 2017 (six other birds). The birds were caught using a whoosh net, by baiting an area around the net until crows were regularly feeding on it and then releasing the net when a family group of crows were present. The birds were housed in small family groups, with the home compartments containing a range of natural enrichment materials, such as logs, branches, shells and pine cones. The sample comprised seven adults, one sub-adult (1–2 years old) and four juveniles (less than 1 year-old), of which seven were male and five female (electronic supplementary material, table S1). Sex was determined by body size, and age by beak coloration [38]. Subjects were generally not food-deprived, and their daily diet consisted of meat, dog food, eggs and fruit, with water available *ad libitum*. The birds were trained to stand on weighing scales (by placing a small piece of food in front of the scale perch) to allow weight to be monitored daily, and all birds were maintained at or above their capture weight during their stay in the aviary. Subjects were tested individually in temporary visual isolation in an adjacent compartment to the rest of the birds. After capture, the birds were first acclimatized to the aviaries in April and then tested in this experiment in May–July. The training and testing procedures for this experiment are outlined in the procedure. All crows were released back into the wild at their site of capture after testing. A previous study indicated that, in a comparable situation to the present study, crows successfully reintegrate into the wild after being subjects in the aviary [39].

### (b) Materials

#### (i) Familiar objects

Birds were first trained with a set of heavy and light objects, as used in [34], with which they were either already familiar (2016 birds), or became familiar with during training (2017 birds). Thus, these are referred to as the ‘familiar objects'. Heavy familiar objects were grey rectangular blocks of varied sizes (approx. 1 × 2 × 3 cm), and red cubes (approx. 2 cm^3^) all weighing 10–15 g. Light familiar objects were white polystyrene rectangular blocks of varied sizes (approx. 1 × 2×3 cm), and blue spheres (1.5–2.5 cm diameter), all weighing less than 1 g.

#### (ii) Novel objects

To test whether birds inferred the weight of objects that they had not previously handled, tests were conducted with unique pairs of visually distinct novel objects, one heavy and one light. Birds had never seen the novel objects before each test session. Heavy novel objects were made from clay and small fishing weights (weighing 10–15 g), and light novel objects were made from polystyrene (all less than 1 g). All objects were covered with paper, tape and/or paint to conceal their construction materials, and varied in size (2–4 cm^3^), shape, colour, pattern and covering material (see electronic supplementary material, figure S1 for examples). One light and one heavy version were made for each object design, and the version presented was counterbalanced across birds (for example, some birds were tested with a *heavy* brown cross, and some birds were tested with a *light* brown cross). This ensured that there were no systematic differences between the appearance of light and heavy objects.

#### (iii) Procedure

**Figure 1.**
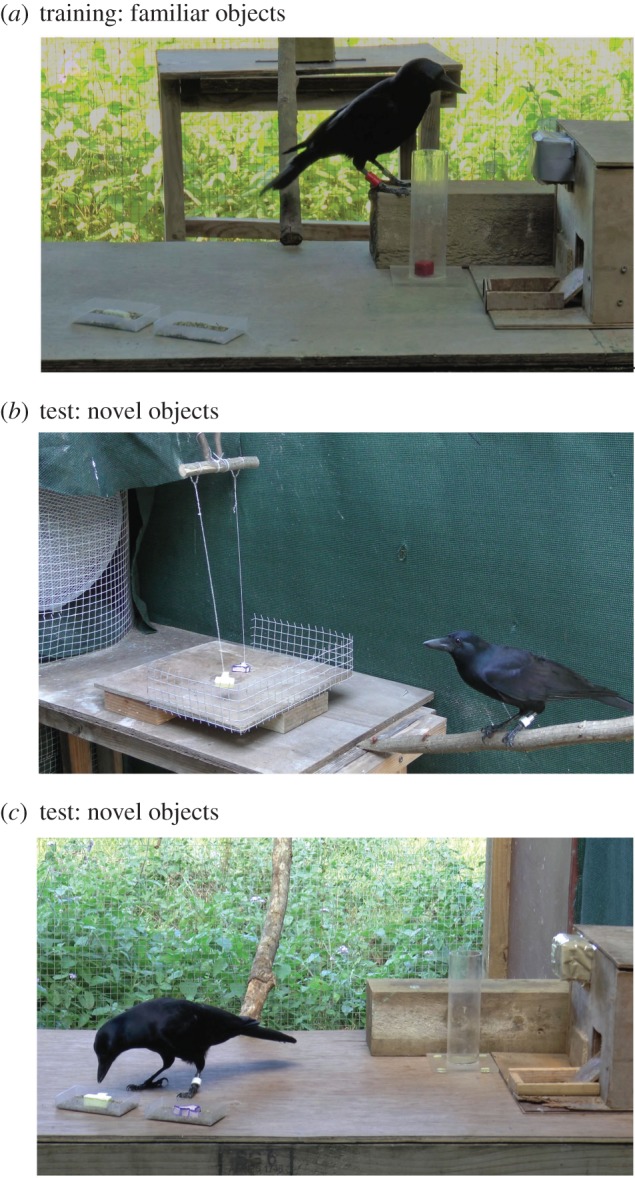
Photographs of the experimental stages. (*a*) Light versus heavy training. Birds learned to drop either familiar light or heavy objects into the tube to obtain rewards (bottle caps containing meat) which slid out of the wooden food dispenser box, controlled remotely by the experimenter from outside the cage. (*b*) Observational phase. Birds observed novel pairs of visually distinct objects (one light, one heavy) presented in front of a fan. The fan was either on, causing the light object to blow around in the breeze (experimental condition) or off and both objects were stationary (control condition). (*c*) Choice phase. The two novel objects were presented on the table close to the tube and food dispenser. Birds were free to interact with both objects and could choose to drop one or both objects into the tube.

*Light* *versus* *heavy training.* All crows were first trained to drop objects into containers to obtain rewards, dropping them into a Perspex apparatus (see [37]), a Perspex tube, or a wooden food dispenser box. 2017 birds were trained to perform this behaviour using natural stones only (weighing 5–15 g), while 2016 birds had participated in two prior experiments that also involved discriminating between some man-made light and heavy objects (see [34,37]). At the start of the present experiment, all birds were trained to drop the familiar objects into a transparent Perspex tube (170 mm high) which was placed next to a wooden box containing an electronic food dispenser. Each time an object was dropped into the tube birds would receive a reward (a bottle cap containing meat) from the dispenser, which was operated remotely by the experimenter (figure 1*a*).

Once birds had learned to drop objects into the tube, they were trained that either only heavy objects (six birds) or only light objects (six birds) were rewarded. For this training, first, on each trial, eight heavy and eight light familiar objects were placed on the table, and birds could drop the objects into the tube to attempt to obtain rewards. Birds received training sessions of this type until they dropped all eight rewarded objects into the tube, before any of the unrewarded objects, on two sessions in a row. The birds dropped all correct objects first from the start. They were then trained to choose between pairs of objects. On each trial, one light and one heavy object were placed on the table, each in a separate sand-filled tray, approximately 50 cm from the tube (figure 1*a*). Birds received eight trials per block, until they chose the correct object on every trial on three blocks in a row (which all birds did from the start of this training stage without error).

To assess whether birds would generalize the rule that only light or only heavy objects were rewarded to novel objects, birds were then given the opportunity to handle a pair of novel, visually distinct objects (one light, one heavy). Each object was placed into a short container, partially filled with sand, on top of a bottle cap containing meat. Birds had to lift each object out of each container (and therefore experience its weight), in order to reach the food. Once birds had lifted each object 14 times they received a test session to determine whether they chose the ‘correct' object (heavy or light depending on prior training) to insert into the tube (see electronic supplementary material for full details). Ten out of twelve birds selected the correct novel object first and dropped this object into the tube. This indicated that, at a group level, these birds were capable of generalizing the rule to novel objects that only heavy or only light objects were rewarded. We therefore progressed to the experiment proper.

*Experiment proper.* In the main experiment, birds received one test per day, with an ‘observation phase' conducted in the morning and a ‘choice phase' conducted in the afternoon, approximately 2–3 h later. There were two conditions: (1) the experimental condition, where the light, but not the heavy, object blew around in a breeze created by an electric fan during the observational phase, and (2) the control condition, where the fan was off, and both objects were stationary during the observational phase. Birds experienced one pair of novel objects per day, alternating between the experimental and control condition each day; half of the birds began with the experimental condition. The novel objects were counterbalanced across the experiment such that all objects were used as stimuli in the experimental condition for some birds and in the control condition for the remainder. This ensured that any differences between our conditions could not be attributed to the objects themselves. Birds tested in 2017 experienced five pairs of novel objects in each condition, while birds in 2016 had three novel object pairs due to time constraints.

*Observational phase.* Beginning in the morning, birds received three observational sessions, each lasting 5 min, spaced approximately 1 h apart. In each observational session, the two novel objects were placed on the table though attached to thin strings suspended from hooks, with the hooks positioned 40 cm above a table, in front of a large electric fan (figure 1*b*). When the fan was on (experimental condition), the light object was light enough to be buffeted around at the end of its string by the breeze the fan created, while the heavy object remained stationary on the table. When the fan was off (control condition), both objects were stationary on the table. To prevent any unintended movement, in the control condition both objects were lightly attached to the table with blue-tack, not visible to the bird. Objects were visible from any point in the cage; however, to ensure the bird gained a close view, and felt the breeze from the fan (when on), in each 5 min trial the experimenter baited a perch that lead to the table on which the objects and fan were presented. The breeze could be felt by a human observer, hence the authors inferred that the birds could feel the breeze, albeit to a degree to which is unknown. At the base of this perch the breeze from the fan (when on) could be felt; however, a 10 cm wire mesh barrier prevented the birds from interacting with the novel objects. The perch was baited three times in each 5 min trial and birds always took the bait.

*Choice phase.* At the start of the choice phase test session, the bird was brought into the testing room, where the same objects and arrangement from the observational phase were already suspended in front of the fan. The fan was either on or off depending on the condition. Unlike the observational sessions, here, the tube, food dispenser box and object trays were also all present on the table. There were three stages in the choice phase: (1) The bird received two familiar object trials to ensure they remembered the rule, which all birds did. (2) The experimenter then baited the perch leading to the suspended objects in the same manner as in the observational trials. (3) Once the bird had obtained the bait, the experimenter entered the room and removed the two novel objects from the strings and placed them on the table, each in a sand-filled tray (figure 1*c*). The bird then had the opportunity to come down to the table and drop one or both objects into the tube. Birds were rewarded in line with the rule they had learned; thus, either the light or heavy object was rewarded. Trials ended when birds left the table after dropping the object(s) into the tube. If the bird did not come down to the table within 1 min, or left the table without interacting with either object, the experimenter baited the table with a small piece of meat. At the end of the trial, the experimenter removed the objects from the tube and placed them back in the sand-filled trays (randomising which object was on the right or left) for a total of five trials.

### (c) Analysis

Our primary measure was which object the bird touched first on its very first trial with each pair of novel objects, before they had any direct experience handling either object. This allowed us to examine whether crows correctly discriminated between the two objects before they had had any direct feedback, via their own manipulation, of each object's weight. We also recorded which object the bird dropped into the tube first on their first trial with each pair of novel objects. For this experiment, the object dropped into the tube first was considered a secondary measurement because once the bird had touched an object—even if only briefly—they had the opportunity to gain extra information about the weight of that object. Thus, the birds' first object touches, for each pair of novel objects, informs us about the birds' understanding of the novel objects when they have observed these objects *only*, while the first object drop occurs after they have observed the objects *and* had some opportunity to interact with the objects. We ran one sample Wilcoxon-signed rank tests—using the percentage of first trials where the object was (a) touched and (b) dropped first, per bird—to assess whether birds touched and dropped the correct, rewarded object first, more often than would be expected by chance. Our control condition was designed to test whether birds used unintended visual cues to identify objects as heavy or light. As we did not know *a priori* how birds would behave in this control condition, we compared performance in both the experimental and control condition to chance.

Additionally, we recorded four further secondary measurements. In our experiment, birds received 5 trials with each pair of objects, therefore, we took four measures of the birds' interactions with the incorrect object during these trials, which we compared across the experimental and control conditions. These additional measurements, collected across all five trials, were the number of trials on which the bird touched the incorrect object (a) first or (b) at any point, and dropped the incorrect object (c) first or (d) at any point (before or after the correct object). We conducted generalized linear mixed models (GLMM) [40] using R (version 3.4.3) [41] to assess which factors influenced our four measurements of interactions with the incorrect object. Interactions with the incorrect object were binary variables, coded as occurring (1) or not (0), and were entered as dependent variables in the models. We included the random effect of bird ID, test number (three tests per condition per bird for 2016 birds, five tests for 2017 birds) and trial number (five trials per test). We included fixed effects of condition (experimental or control condition) and rewarded object (whether the bird was rewarded for light or heavy objects). We used likelihood ratio tests to compare the full model (all predictor variables, random effects and control variables) first with a null model (random effects, no predictor variables), and then with reduced models to test each of the effects of interest [42]. All experimental trial videos were coded by S.A.J. and Anna Frohnwieser, finding 100% agreement.

## 3. Results

**Figure 2.**
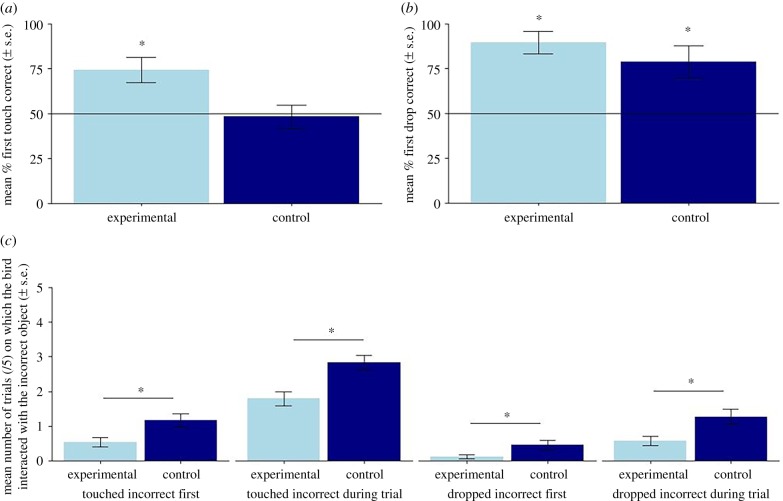
The mean percentage of tests on which the birds' very first (*a*) touch and (*b*) drop was of the correct object (light or heavy). Line denotes chance levels of 50%. The birds touched the correct object first significantly more than expected by chance in the experimental, but not control condition, and dropped the correct object first significantly more than chance in both conditions. (*c*) Mean number of trials, out of five, on which the bird touched the incorrect object first or at all, and dropped the incorrect object first or at all. For each measure, the birds interacted with the incorrect object significantly more often in the control condition than experimental condition.

Our primary finding was that birds touched the correct object first, on their first trial with each pair of novel objects, significantly more often than chance in the experimental condition (35/48 first touches correct, 72.9%, one-sample Wilcoxon test: *Z* = 70, *p* = 0.01). Birds performed at chance level in the control condition when the fan was off and both objects were stationary (23/48, 47.9%, *Z* = 27, *p* = 0.34; figure 2*a*). When examining the very first novel object test that the birds received in each condition, 11/12 birds touched the correct object first in the experimental condition (binomial test: *p* = 0.003); 6/12 birds touched the correct object first in the control (*p* = 0.226).

Birds were highly accurate when choosing the correct object to drop on their first trial with each pair of novel objects in both conditions (experimental: 44/48 first drops correct, 92%, one-sample Wilcoxon test: *Z* = 77, *p* = 0.002; control: 38/48, 79%, *Z* = 68.5, *p* = 0.017; figure 2*b*). Across both conditions, birds almost exclusively dropped the correct object first into the tube if they had touched the correct object first (experimental: 35/35 trials, 100%; control: 22/23, 96%), but also dropped the correct object first on more than half of the trials where they initially touched the incorrect object (experimental: 9/13, 69%; control: 16/25, 64%).

For the models analysing the birds' interactions with the incorrect objects across all trials, the null model and the full model differed significantly for all four variables of interest, finding significant effects of condition on each variable: (1) *Touched incorrect first* (*χ*^2^ = 15.758, d.f. = 2, *p* ≤ 0.001; Experimental or Control condition: *z* = 3.716, *p* ≤ 0.001); (2) *Touched incorrect at any point* (*χ*^2^ = 27.747, d.f. = 2, *p* ≤ 0.001; *z* = 4.679, *p* ≤ 0.001) (3) *Dropped incorrect into the tube first* (*χ*^2^ = 15.76, d.f. = 2, *p* ≤ 0.001; *z* = 3.72, *p* ≤ 0.001) and (4) *Dropped incorrect at any point* (*χ*^2^ = 20.278, d.f. = 2, *p* ≤ 0.001; *z* = 4.098, *p* ≤ 0.001). For all four variables, the birds interacted with the incorrect object more often in the control than in the experimental condition (figure 2*c*). There was no significant effect of ‘rewarded object' (whether the light or heavy object was rewarded) on any of the variables of interest (*p* > 0.05).

## 4. Discussion

Our results demonstrate that New Caledonian crows were significantly more accurate at discriminating between novel light and heavy objects when they had previously observed these objects either moving or remaining stationary in a breeze created by an electric fan. After this experience, birds touched the correct object first, on their very first trial with each set of novel objects, more often than expected by chance. When the fan was off—and birds did not have access to this source of information about the objects' weight—the birds were equally likely to touch the incorrect or correct object first.

Our results appear to be robust for a number of reasons. First, there were no group difference in birds previously trained to select light objects versus heavy objects. Consequently, our results cannot be explained by a general preference for selecting the objects that moved (or did not move) in front of the fan: each group preferentially selected the object which fitted the rule that they had learned. Second, all the novel objects were used in both control and experimental conditions (counterbalanced across birds). Given that birds performed at chance in the control condition, they did not appear to be able to use any unintended visual cues from the objects to determine which object was heavy or light. Third, birds received a small number of tests to limit opportunities for learning. Importantly, 11/12 birds touched the correct object first on their very first trial in the experimental condition versus 6/12 in the control. These first trial successes demonstrate that the birds did not succeed by learning to use movement as a cue over the course of the experiment. With these factors in mind, the simplest explanation for our results is that New Caledonian crows used pre-existing knowledge about the behaviour exhibited by differently weighted objects in the wind, to infer whether the novel objects were heavy or light, then used this information to guide their choices.

Importantly, the information birds used could not have been a single assessment of utility for the task (e.g. whether the object was ‘good to drop into the tube'). Rather, there was a single underlying property (weight) that predicted both the object's utility in the task, and the object's movement in a breeze. Only an inference of a single underlying property would have allowed them to transfer information observed *allocentrically*—how the wind affected different objects—into an egocentric choice between two objects of different weight [43,44]. However, the exact content of this abstract concept of ‘weight' does remain unclear. Crows may have had a full understanding of weight like humans; alternatively, they may have possessed only a partial understanding of weight. For example, the crows may have evaluated the objects in terms of how easily they could be moved, both by the wind and by themselves (i.e. something akin to displaceability), but may not have evaluated the objects as being ‘heavy' or ‘light' in terms of other physical interactions, such as whether they would also sink or float. Another possibility is that their understanding of weight may have been based on an ability to perceive the affordances of objects by observing the wind's effect on them [45]. Testing to see how closely the crows' concept of weight across different contexts mirrors that of humans will be a focus of future work.

Across all five choice trials with each novel object pair, birds interacted with the incorrect object more in the control than the experimental condition. However, despite the difference in the number of interactions with the incorrect object across the two conditions overall birds made relatively few errors after their first touch in both conditions (figure 2*c*). This occurred even though there was no penalty to the bird for interacting with both objects during test trials, and birds were not required to drop the correct object first to obtain the reward. When they did touch the incorrect object first on their first trial, birds successfully switched to dropping the correct object into the tube for their first object drop in over half of these trials, indicating that they could typically discard the incorrect object without receiving direct feedback on whether this particular object was rewarded. This did not happen on every trial, possibly due to limits on the birds' inhibitory control [34]. However, overall, this pattern of behaviour demonstrates that these birds were highly capable of retaining and generalizing the rule that only light or only heavy objects were rewarded, and needed minimal handling experience to learn and remember which of the novel objects was light or heavy across the five trials of each novel object test. The weight of small, manipulable objects may therefore be a particularly salient characteristic for this species to learn about [46].

A number of species select nuts and seeds on the basis of their weight before attempting to open them [13–16]. New Caledonian crows eat candlenuts and snails, which they drop from heights on to hard surfaces to break open [47,48]. It has not been tested whether they select candlenuts on the basis of their weight, though our observations indicate that this would be possible. The weight of candlenuts and snails, similar in size to our objects, may be an ecologically relevant feature, and may account for the birds' rapid learning, and inferential abilities, when tested with these types of objects.

Here, we were interested in whether these birds could infer the weight of the novel objects, not whether the birds *remembered* which object had moved in the breeze. Therefore, our experimental design minimized memory demands and facilitated encoding of the relevant object properties. We presented the test set-up before, after and concurrently with the opportunity to observe the novel objects (see [49] for task presentation order effect on information encoding in a primate tool-use task). Equally, here, we presented birds with three 5 min trials of continuous motion (or absence of motion) to ensure they had sufficient time to observe the events. Having established that birds do gain information from observing objects moving in the wind, it may be of interest to know how much observational time is necessary. In human infants, the level of exploratory interactions required by infants to learn about object properties and relation differs across tasks [50]. For example, 11-month-olds need to explore task materials before they will demonstrate that they can infer an object's weight using compression information [8]. Crows may make rapid judgements about object weight from only brief observations of the objects' movements, or learn from single events, such as the sound it makes when dropped. Future experiments are required to test this.

Our findings are particularly interesting as they provide the first indication that a bird species has the capacity to make inferences about weight. This finding, though testing only a single type of inference, contrasts with Povinelli's claim, based on the failures exhibited by chimpanzees, that only humans have a causal understanding of weight [9]. Here, New Caledonian crows demonstrated first trial successes, followed by continued above chance performance, in a novel situation which provided only indirect information about the objects' weights. Thus, these birds appear to have general prior expectations of how differently weighted objects should behave when observed in a novel context.

These results stand in contrast to recent findings suggesting that New Caledonian cannot use observations of physical interactions ‘accidentally' resulting from their own actions to perform causal interventions [51]. In the present study, crows were able to pick-up causal information from observing the wind interacting with an object. Among humans, the extraction of object features, like weight, from observing another person's actions appears to involve implicit, rather than explicit, reasoning and involves the observer's motor system. That is, the observer's own prior experiences of handling objects can inform their perception of objects, when they are seen to be handled by another individual [52,53]. If a similar cognitive process underpinned the crows performance here, it could explain this discrepancy: New Caledonian crows are capable of implicitly inferring causal information, but not explicitly using this to create novel behaviours. Another possibility is that the crows failure was a task artefact [51]. When confronted with an artificial apparatus whose mechanism is not easily comprehensible, the birds may revert to basic instrumental learning of action-outcome associations despite being capable of more sophisticated reasoning in more ecologically relevant contexts. While the fan used in this experiment was also artificial, and both studies involved an ecologically relevant action (dropping)—though the goal of this action differed—attending to the presence of wind would likely have been more ecologically salient than attending to the inner workings of a food dispenser. Such an explanation requires further investigation.

New Caledonian crows have also failed to demonstrate an immediate understanding of the relevance of weight in other contexts; particularly, experiments involving artificial apparatuses [36,37]. When presented with a Perspex apparatus containing a collapsible baited platform, crows drop feathers [36], or other light objects [37], which were insufficiently heavy to collapse the platform and obtain the reward. It should be noted that dropping the light objects did not prevent the crows from obtaining the reward as they could still drop heavy, rewarded objects afterwards. This performance is similar to the equally unsuccessful behaviours in a comparable task with chimpanzees [9]. In the light of our current results, these failures may indicate limitations in the animals' understanding of the apparatuses, rather than a generally poor grasp of the concept of weight. Additionally, as the crows also sometimes dropped unrewarded (light or heavy) objects in the present study, this behaviour generally may reflect play or exploration, rather than an inability to infer the weight of objects based on their movement. Future studies may address this by ending the trial after the subject has made one choice.

To date, the ability to learn from observations has almost exclusively been studied in a social context. Typically, subjects observe another individual's actions or interactions with an object, and are then tested behaviourally to determine what they have learned (e.g. [1,54]). However, New Caledonian crows have not performed well in cognitive tests conducted in a social context, such as observing demonstrators. Like many non-primates, without extensive training, these birds fail to choose the rewarded cup after observing a human visibly place a reward into one of three containers [55]. They have also failed to recognize the need for a partner in a cooperative task [56], and demonstrate stimulus enhancement, but not imitation, in a social learning task [57]. Our results are striking in that they suggest that New Caledonian crows are capable of gaining nuanced information about objects from observing their *physical* interactions, despite their poor performance on tasks that involve observing other types of events.

Overall, our results suggest that these birds are capable of making inferences about the properties of objects in the world around them, without having to directly experience those properties themselves. This form of learning through observations has received minimal attention among animals to date, but may reflect an important source of information. Failures by chimpanzees on comparable tasks have led some researchers to argue that only humans are capable of representing weight as a causal mechanism [9]; however, our results suggest this is not the case. Determining the weight of small objects may be particularly relevant to New Caledonian crows, as they consume candlenuts, for which weight correlates with quality, and drop snails, which can be very heavy relative to a crows' body weight [48]. They also use tools, where the weight of a tool is likely to affect effort and outcome, which may lead crows to be more selective. Whether this ability to infer the weight of objects is widely spread across the animal kingdom, or only a feature of animals with broadly high levels of physical cognition, is presently unknown.

## Supplementary Material

Supplementary Methods and Tables S1

## Supplementary Material

Supplementary Dataset

## Supplementary Material

Supplementary Figure S1

## Supplementary Material

Supplementary Movie S1

## Data accessibility

The data are accessible in the electronic supplementary material.
