# Supplementary material for: New Caledonian crows infer the weight of objects from observing their movements in a breeze
Source: Proc Biol Sci. 2019 Jan 9;286(1894):20182332. doi: 10.1098/rspb.2018.2332 (PMC6367178; doi:10.1098/rspb.2018.2332)
Supplement: Supplementary Methods and Tables S1 [file rspb20182332supp1.pdf]

Supplementary Methods for: New Caledonian crows infer the weight of objects from observing their movements in a breeze

Supplementary Methods

Handling Experience

After learning the rule that only light or only heavy objects were rewarded, but before progressing to their first experimental or control test, all birds completed a handling experience phase. This was designed as a pre-test to determine whether birds appeared to generalise the learnt rule to novel objects of known weights. The procedure was as follows.

Handling Phase: Birds were given the opportunity to handle two visually distinct novel objects: one light and one heavy. The novel objects used in this test were selected from the same set of objects as those used in the main experiment (examples in Fig. S1). Each bird was tested using a different object pair. First, birds were habituated to the objects. The experimenter placed the two objects onto the table and baited underneath and on top of each object with scraps of meat. This was repeated twice for each bird. Then, each novel object was placed into a short container, partially filled with sand, on top of a bottle cap containing meat (Fig. S2, below). Birds had to lift each object out of each container (and therefore experience its weight), in order to reach the reward. Birds received 14 trials split across two sessions, conducted on the same or consecutive days, where they removed both objects from containers. Once completed birds moved on to a choice phase.

Choice Phase: The choice phase was conducted in the same manner as the experimental and control conditions. At the start of the choice phase session, the bird was brought into the testing room. The tube, food dispenser box and object trays were present on the table, and the session began by giving each bird two familiar object trials to ensure they remembered the rule, which all birds did. The experimenter then entered the room and removed all the apparatus from the table. For the second stage of the session, the experimenter placed the two sand-filled containers used in the handling phase on the table, each containing one of the novel objects on top of a bottle cap containing meat. The bird was required to extract each novel object from each container on two consecutive trials, as they had done in the handling phase. Immediately after this they received the third stage of the test: the choice trials. The experimenter re-entered the room, collected the novel objects and containers, and then replaced the test apparatus (the tube, food dispenser box and object trays) on the table. The experimenter placed each of the novel objects into the object trays. The bird had the opportunity to come down to the table and drop one or both objects into the tube. Identically to the main experiment, trials ended when birds left the table, or when both objects were

no longer accessible (because they had been dropped into the tube or off the table). If the bird did not come down to the table within 1 minute, or left the table without interacting with either object, the experimenter baited the table with a small piece of meat. At the end of the trial, the experimenter removed the objects from the tube and placed them back in the sand-filled trays (randomising which object was on the right or left) for a total of 5 trials.

Results: 10/12 birds touched the correct, rewarded object first on their first trial. Therefore, the experimenter progressed to the main experiment.

Across all five trials of the test, on average, birds touched the incorrect object first on 0.4/5 trials, touched the incorrect object at any point on 3.75/5 trials, dropped the incorrect object into the tube first on 0.25/5 trials and dropped the incorrect object into the tube at any point on 3/5 trials. Thus, birds were highly accurate at touching and dropping the correct object *first* on each of the five trials of the test, but also then interacted with incorrect object on the majority of trials.

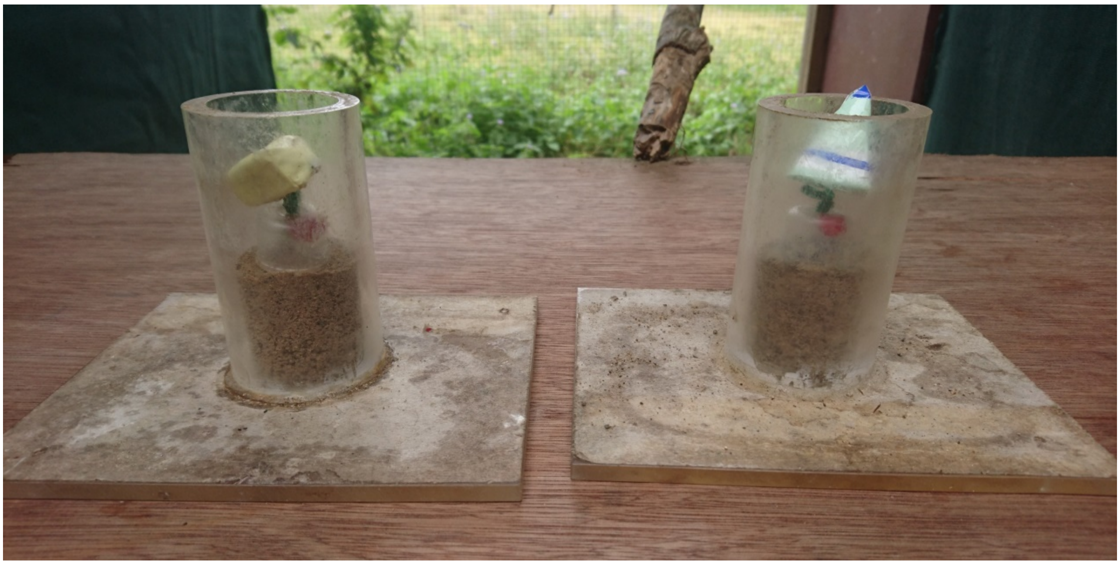

Figure S2: Photo of the handling phase set-up.

Supplementary Table S1: Subject Information

					Experimental Condition		Control Condition	
**Bird**	**Sex**	**Age**	**Rewarded**	**Test Year**	First touches correct	%	First touches correct	%
Elvis	M	Adult	Light	2017	3/5	60	3/5	60
Freddie	M	Juvenile	Light	2017	4/5	80	1/5	20
Janis	F	Adult	Light	2017	4/5	80	2/5	40
Noir	F	Adult	Light	2016	3/3	100	1/3	33
Sort	F	Adult	Light	2016	1/3	33	3/3	100
Svart	M	Sub-adult	Light	2016	3/3	100	1/3	33
Annie	F	Juvenile	Heavy	2017	3/5	60	3/5	60
Bonnie	F	Juvenile	Heavy	2017	5/5	100	2/5	40
David	M	Adult	Heavy	2017	4/5	80	3/5	60
Black	M	Juvenile	Heavy	2016	3/3	100	1/3	33
Nero	M	Adult	Heavy	2016	2/3	66	2/3	66
Preto	M	Adult	Heavy	2016	1/3	33	1/3	33
