# Supplementary figures and images for: New Caledonian crows infer the weight of objects from observing their movements in a breeze
Source: Proc Biol Sci. 2019 Jan 9;286(1894):20182332. doi: 10.1098/rspb.2018.2332 (PMC6367178; doi:10.1098/rspb.2018.2332)

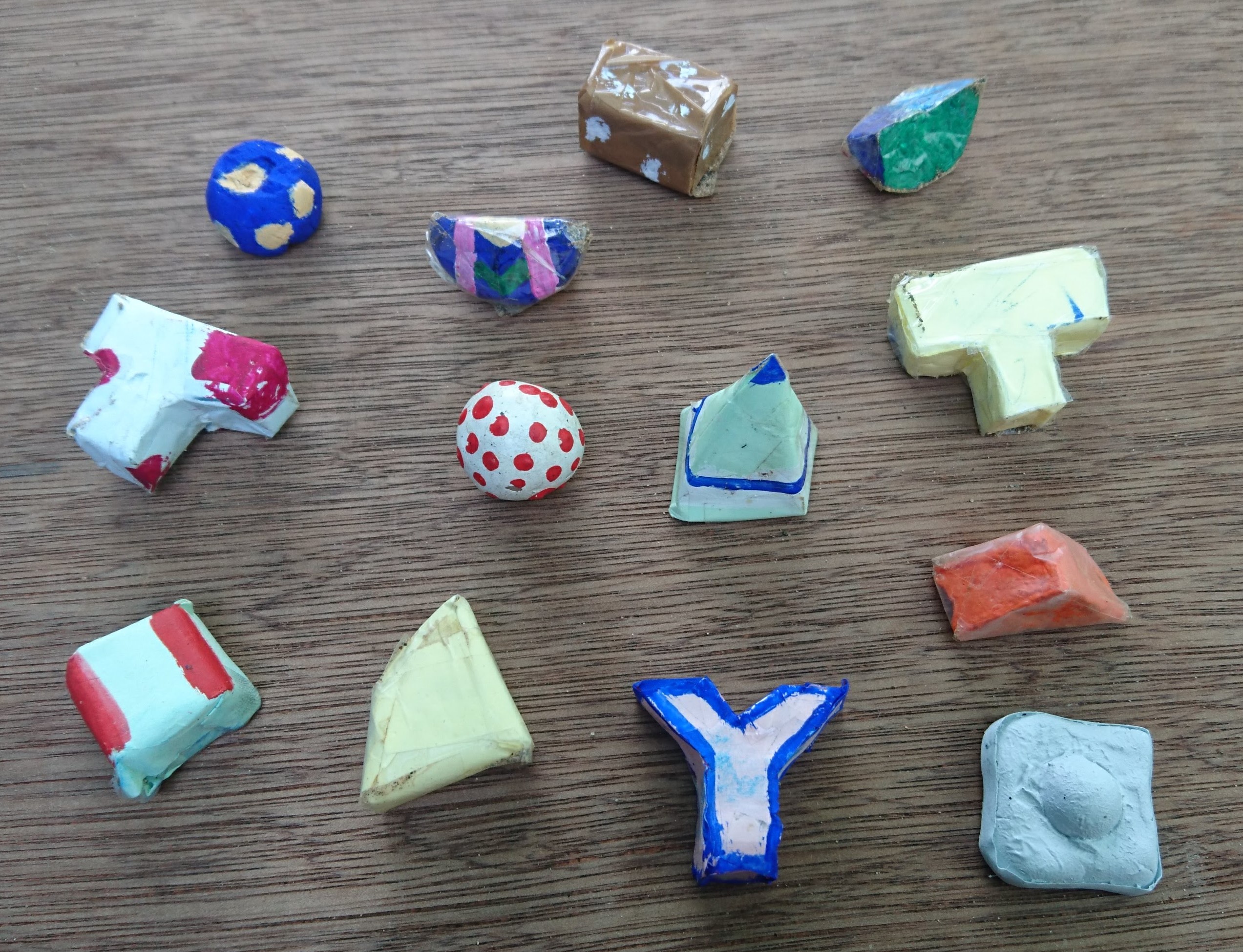

Supplement: Supplementary Figure S1 [file rspb20182332supp3.jpg]
